# Supplementary material for: Applying user-centered design to enhance the usability and acceptability of an mHealth supervision tool for community health workers delivering an evidence-based intervention in rural Sierra Leone
Source: Glob Ment Health (Camb). 2025 Apr 11;12:e67. doi: 10.1017/gmh.2025.38 (PMC12231310; doi:10.1017/gmh.2025.38)
Supplement: Antonaccio et al. supplementary material [file S205442512500038Xsup001.pdf]

A **user-centered design** (UCD) approach can **promote the usability and acceptability of mHealth tools** to *enhance the delivery of evidence-based interventions* in low-resource settings.

Context:
In Sierra Leone, child mortality rates remain high, and physical abuse and maltreatment are prevalent. This study used a UCD approach to develop and evaluate an mHealth supervision tool for CHWs delivering the Family Strengthening Intervention for Early Childhood Development plus Violence Prevention (FSI-ECD+VP) for families in rural Sierra Leone.

1. ANALYZE

Brief **surveys**, **focus group discussions** (FGDs) with supervisors and CHWs to identify supervision *challenges* and *needs*.

2. DESIGN

Prototype the mHealth tool based on analysis findings—including *content*, *progress tracking*, and *fidelity checklist* features.

3. DEVELOP

Refine mHealth supervision tool via iterative ***user testing***, gathering feedback on usability and making necessary adjustments.

4. IMPLEMENT

Pilot the mHealth tool with CHWs and supervisors *during delivery of the FSI-ECD+VP* intervention.

5. EVALUATE

Examine **usability** of the mHealth tool through *mixed methods evaluation;* identify **strengths** and areas for **improvement**

The analysis phase revealed challenges like a need for **improved documentation**, **data collection**, and **communication**, which the mHealth tool was designed to address.

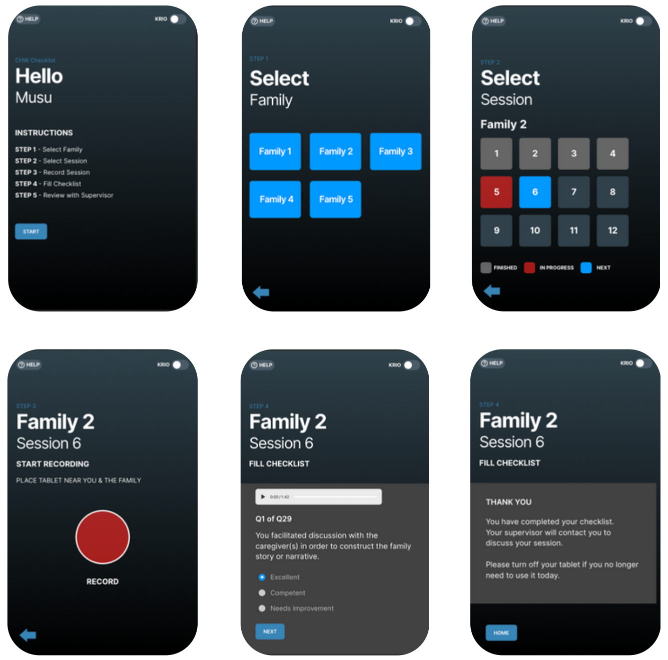

A mixed methods evaluation found that the mHealth tool was **well-received** and perceived as *easy to use* and *helpful*. Future studies should explore the ***scalability*** and ***sustainability*** of mHealth tools in similar settings and for other evidence-based interventions.

Advantages of Visual Charts

Convey a great number of information in an effective manner

Simplifies statistics

Use visual charts to communicate info more effectively.

Easy to understand

Use visual charts to communicate info more effectively.

Adds credibility

Use visual charts to communicate info more effectively.

Makes an impact

Use visual charts to communicate info more effectively.

RESOURCE PAGE

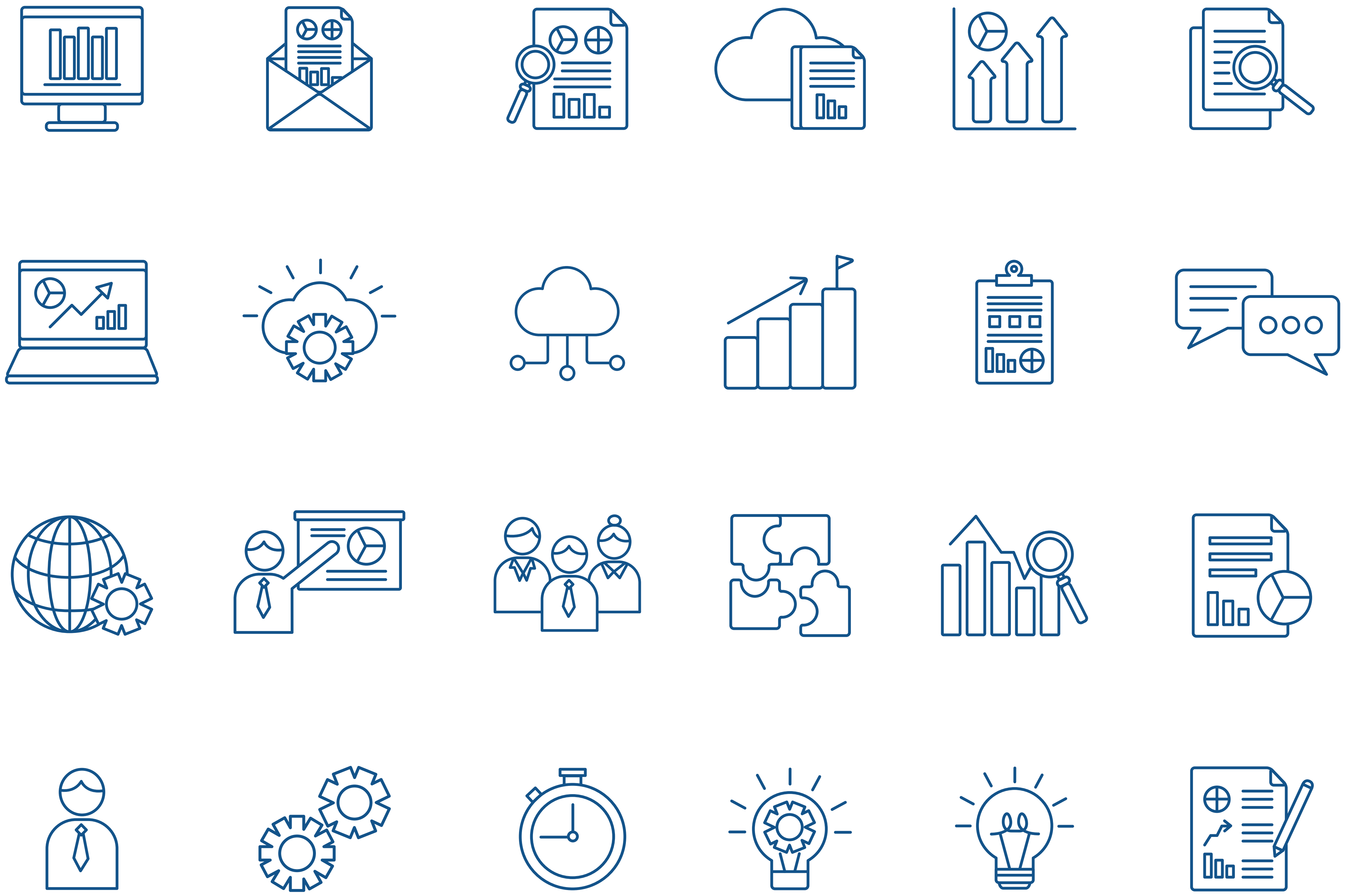

Use these design resources in your Canva Presentation. Happy designing!

Don't forget to delete or hide this page before presenting.

WAVE'S END SWIMWEAR

5-Step Ordering Process

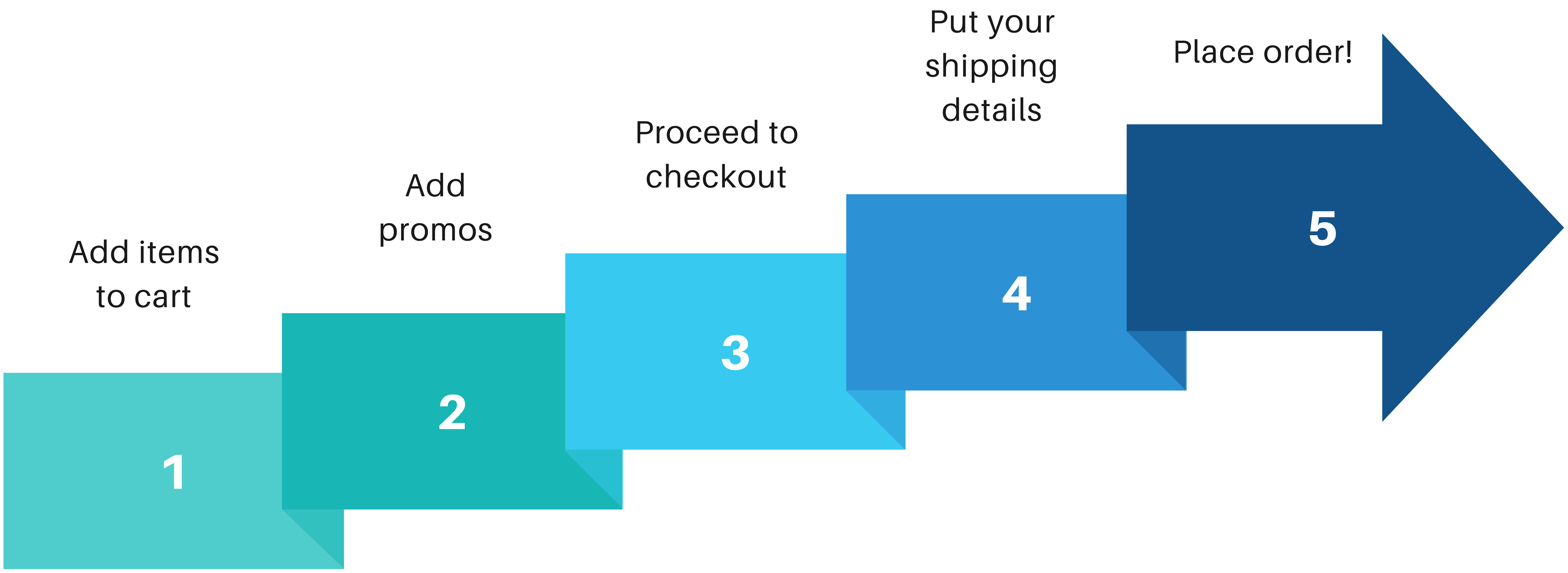

Stages of the UCD Process

1. ANALYZE	2.	HYPOTHESIZE	RESEARCH	CONCLUSION
Identify a problem and form a thesis statement.	Review literature related to your topic.	Come up with an educated guess based on your research.	Read resources to support your hypothesis.	Interpret the results and write your conclusions.

RESOURCE PAGE

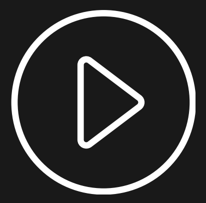

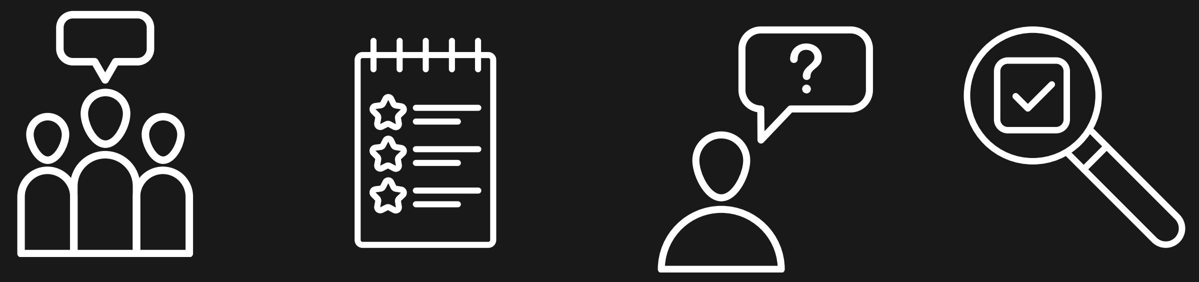

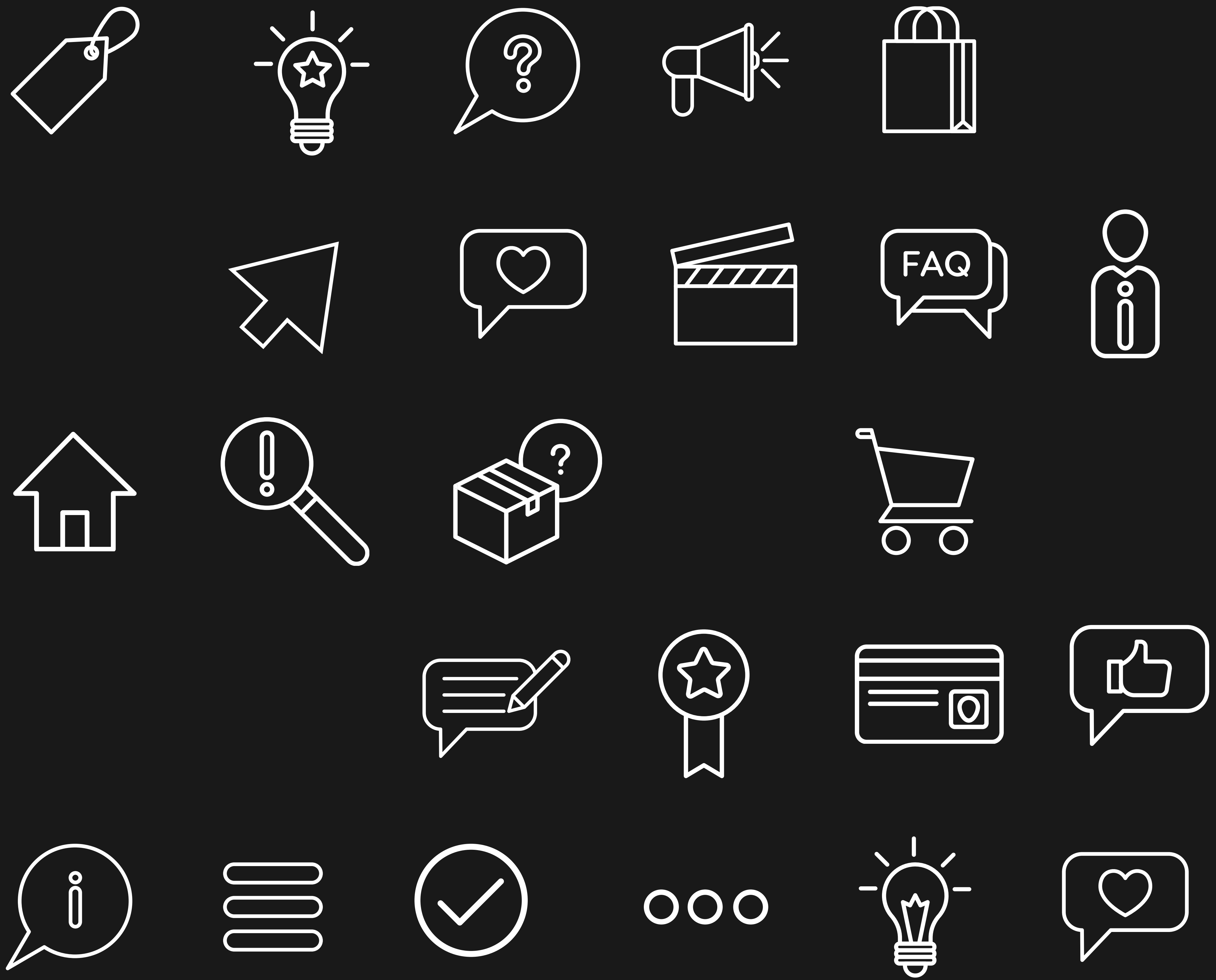

Use these design resources
in your Canva Presentation.
Happy designing!

Don't forget to delete or hide this
page before presenting.
